# Biome stability predicts population structure of a southern African aridland bird species

**DOI:** 10.1002/ece3.6175

**Published:** 2020-03-27

**Authors:** Guinevere O. U. Wogan, Gary Voelker, Graeme Oatley, Rauri C. K. Bowie

**Affiliations:** ^1^ Department of Integrative Biology Museum of Vertebrate Zoology University of California Berkeley CA USA; ^2^ Department of Wildlife and Fisheries Sciences Biodiversity Research and Teaching Collections Texas A&M University College Station TX USA; ^3^ Department of Geography College of Life and Environmental Sciences University of Exeter Exeter UK; ^4^ DST/NRF Centre of Excellence at the Percy FitzPatrick Institute University of Cape Town Rondebosch South Africa

**Keywords:** Anthropogenic change, ephemeral speciation, landscape genetics, refugia

## Abstract

Environments are heterogeneous in space and time, and the permeability of landscape and climatic barriers to gene flow may change over time. When barriers are present, they may start populations down the path toward speciation, but if they become permeable before the process of speciation is complete, populations may once more merge. In Southern Africa, aridland biomes play a central role in structuring the organization of biodiversity. These biomes were subject to substantial restructuring during Plio‐Pleistocene climatic fluctuations, and the imprint of this changing environment should leave genetic signatures on the species living there. Here, we investigate the role of adjacent aridland biome boundaries in structuring the genetic diversity within a widespread generalist bird, the Cape Robin‐chat (*Cossypha caffra*). We find evidence supporting a central role for aridland biomes in structuring populations across Southern Africa. Our findings support a scenario wherein populations were isolated in different biome refugia, due to separation by the exceptionally arid Nama Karoo biome. This biome barrier may have arisen through a combination of habitat instability and environmental unsuitability, and was highly unstable throughout the Plio‐Pleistocene. However, we also recovered a pattern of extensive contemporary gene flow and admixture across the Nama Karoo, potentially driven by the establishment of homesteads over the past 200 years. Thus, the barrier has become permeable, and populations are currently merging. This represents an instance where initial formation of a barrier to gene flow enabled population differentiation, with subsequent gene flow and the merging of populations after the barrier became permeable.

## Graphical abstract

This study investigates the landscape genetics of a Southern African bird species across the aridland biomes. We evaluate population structure and demography in combination with species and biome modeling to make explicit connections between the changing aridland landscape and genetic patterns. We find evidence for an ephemeral barrier among these biomes, with one of the aridland biomes serving as a barrier to gene flow for birds in isolated biome refugia, but that Anthropogenic change within this barrier (the establishment of homesteads) has made the barrier permeable enabling recurrent gene flow.

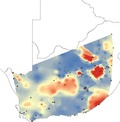

## 1 INTRODUCTION

It is well documented that spatial heterogeneity plays a role in how organisms disperse across habitats and landscapes, which in turn shapes patterns of gene flow (Manel, Schwartz, Luikart, & Taberlet, 2003). Yet, habitats and landscapes are rarely, if ever, temporally static entities. As such, dynamic climate‐mediated changes to habitats, landscapes, and even entire biomes have the potential to modulate dispersal and gene flow through time (Fritz et al., 2013; Jetz & Fine, 2012). Barriers to dispersal can include relatively static geographic features such as rivers or mountains, or potentially more permeable barriers such as habitats that vary in their degree of inhospitably through time; but in either case, the temporal persistence of such dispersal barriers can lead populations to diverge into separate species. Barriers vary in permeability to gene flow over time, that is, they can be ephemeral. As such, they may start populations on the path toward speciation while present, but once removed can lead to the formation of hybrid zones, genetic introgression, or the merging of formerly distinct lineages, depending on how far along the speciation continuum those (formerly) isolated populations have traversed (Rosenblum et al., 2012; Seehausen, 2006).

Habitat refugia and ephemeral habitat barriers were formed by the climate fluctuations characterizing the Plio‐Pleistocene. For species endemic to a particular biome, it can be hypothesized that the history of the biome will be reflected in the demographic history of the species. This has been demonstrated for both glacial and habitat refugia, with glacial refugia having shaped the distribution of diversity in the Northern Hemisphere (Hewitt, 2004), and habitat refugia, in particular tropical forest refugia, having served as important harbors for species diversity and genetic diversity in the Australian Wet Tropics (Graham, Moritz, & Williams, 2006), the Brazilian Atlantic Forests (Carnaval, Hickerson, Haddad, Rodrigues, & Moritz, 2009), and the lowland and montane forests of Africa (e.g., Barratt et al., 2018; Fjeldså & Bowie, 2008; Huntley, Keith, Castellanos, Musher, & Voelker, 2019; Portik et al., 2017; Voelker, Outlaw, & Bowie, 2010). However, for widespread habitat‐generalist species, the role of habitats and habitat refugia in structuring diversity is less certain, since by definition, these species are not limited to a particular habitat type and possess traits that allow them to utilize a range of habitats and resources. Does the distribution of biomes shape the population genetic structure and demographic history of widespread generalist species that are able to traverse habitat and climate space with ease? For highly mobile generalist species, we expect to find little genetic differentiation across their range, and generally predict a pattern of isolation by distance, with the caveat that geographic distance alone may be insufficient to drive diversification in the absence of discrete barriers to gene flow (Latch, Reding, Heffelfinger, Alcalá‐Galván, & Rhodes, 2014; but see Irwin, 2003).

**Figure 1:**
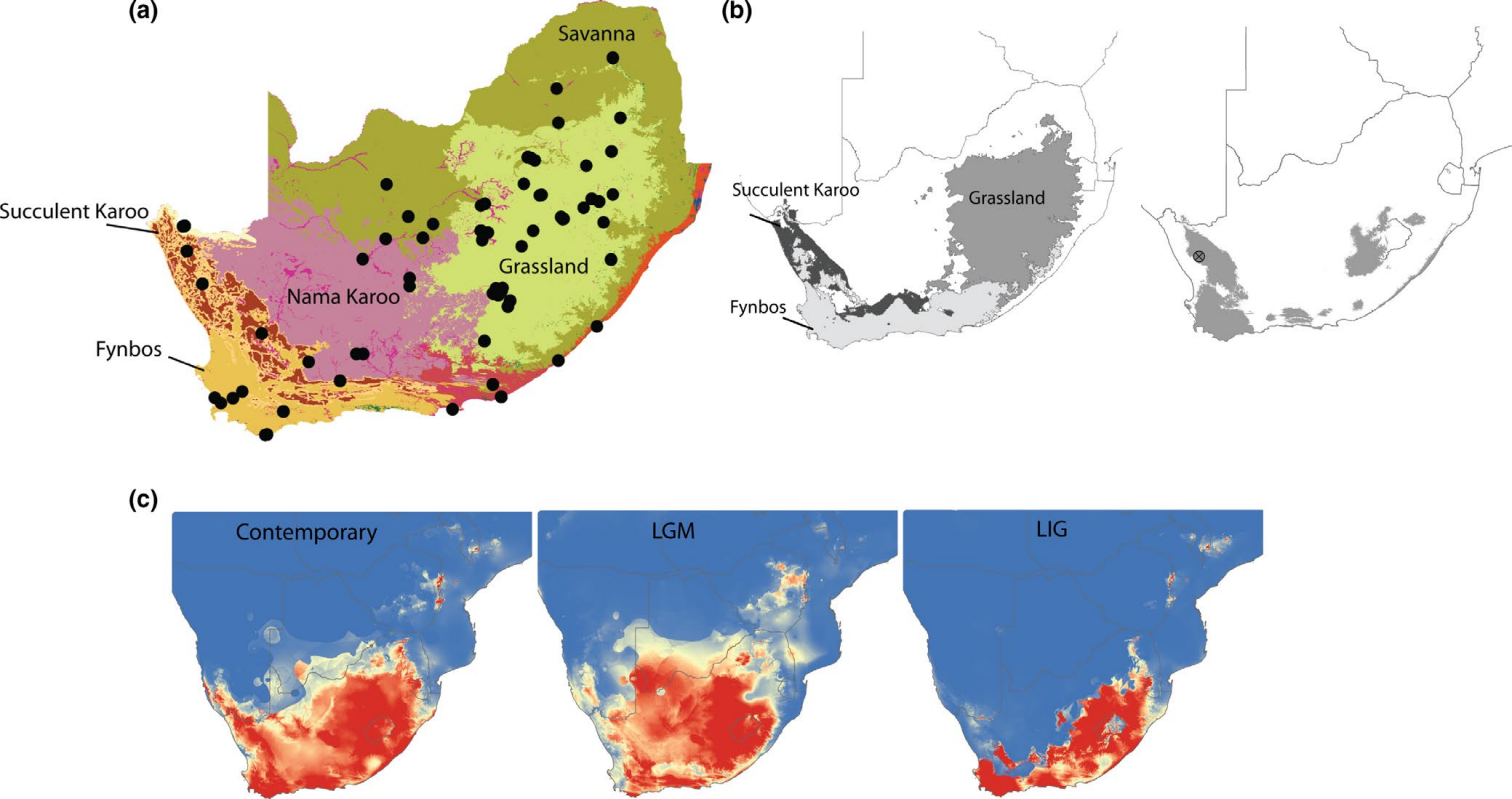
Maps depicting (a) sampling localities (*n* = 82) included in this study overlain on the contemporary distribution of South African biomes. (b) Biome refugia (left) and species refugia (right) modeled using environmental niche models. (c) Environmental niche models for *Cossypha caffra* modeled using the contemporary distribution and projected to the last glacial maximum (LGM) ~18–21 ky before present and the last interglacial period (LIG) ~120–140 ky before present. Environmental suitability is scaled from red (high suitability) to blue (low suitability)

While the interplay between species, genetic diversity, and biome history of Afro‐tropical forests has been fairly well‐studied, similar studies of species occupying the diverse aridland biomes which dominate the continent have lagged behind. At a broad scale, it is clear that pan‐African savanna biomes have a demonstrated effect on the population structure of several large ungulate mammal species with wide distributions as well as bird taxa (e.g., Alpers, van Vuuren, Arctander, & Robinson, 2004; Fuchs, Crowe, & Bowie, 2011; Fuchs, Fjeldså, & Bowie, 2017; Fuchs, Swardt, Oatley, Fjeldså, & Bowie, 2018; Rakotoarivelo, O’Donoghue, Bruford, & Moodley, 2019; Smitz et al., 2013). However, the role of more regional aridland biomes in structuring diversity is less understood. Southern Africa, for example, is well known for its high diversity of aridland biomes (Figure 1a; Mucina & Rutherford, 2005), each with different characteristics and each with high levels of floristic and faunistic endemism (Colville et al., 2014; Cowling, Rundel, Desmet, & Esler, 1998; Richardson et al., 2001). Differing rainfall patterns across the region influence the aridland biomes, with much of the Nama Karoo, Fynbos, and Succulent Karoo biomes subject to winter rainfall, while the Savanna and Grassland biomes receive summer rains (Mucina & Rutherford, 2005). Over the past five million years, the Benguela current along the western coast of Southern African has strengthened (Diester‐Haass, Meyers, & Vidal, 2002) bringing greater aridity to the neighboring terrestrial coastal and interior habitats (Shi, Dupont, Beug, & Schneider, 2000). This increase in aridity, coupled with the uplift of the interior escarpment during the Pliocene (Partidge, 1997), has enabled a steep west‐to‐east gradient in aridity to develop, with the interior escarpment being dryer than the coasts.

The high levels of endemism in South Africa suggest that biome boundaries have played a pivotal role in speciation, through the modulation of gene flow and diversification among biomes for several mammal and reptile taxa (Du Toit, Vuuren, Matthee, & Matthee, 2012; Russo, Chimimba, & Bloomer, 2010; Tolley, Bowie, Measey, Price, & Forest, 2014; van Rensburg, Levin, & Kark, 2009). Furthermore, recent GIS‐based analyses for Southern African birds suggest that aridland refugia may have played an important role in shaping macroecological diversity patterns in and among regional biomes (Huntley et al., 2016; Huntley, Midgley, Barnard, & Valdes, 2014). Despite this biome‐genetic diversification relationship, few empirical studies have evaluated the role of temporal habitat stability (i.e., habitat persistence through time) in structuring genetic diversity. Most genetic studies on South African aridland birds have focused on species complexes where parapatric speciation across biome boundaries has given rise to habitat specialist species (Oatley, Voelker, Crowe, & Bowie, 2012; Ribeiro, Lloyd, & Bowie, 2011; Ribeiro, Lopes, & Bowie, 2012; Ribeiro et al., 2019).

Discrete barriers are far less likely than biome boundaries to impact highly mobile birds. For example, the widespread generalist Fiscal Shrike (*Lanius collaris*) consists of a single genetically homogenous lineage distributed across the entire southern African region (Fuchs et al., 2011). However, discrete geographic barriers have impacted dispersal‐limited southern African vertebrate taxa such as small mammals and reptiles (e.g., Barlow et al., 2013; Portik, Bauer, & Jackman, 2011; Tolley, Makokha, Houniet, Swart, & Matthee, 2009; Tolley et al., 2009; Willows‐Munro & Matthee, 2011). When substructure is present among vertebrate taxa, it can either reflect historical barriers to gene flow or contemporary ecological factors (da Silva & Tolley, 2017), but we know little about these factors with respect to Southern African bird lineages.

Here, we evaluate genetic diversity in light of contemporary ecological landscape features, and deeper time biome stability for the Cape Robin‐chat (*Cossypha caffra*), a widely distributed, aridland generalist bird species. There are two morphologically distinct subspecies in South Africa: *Cossypha caffra caffra* which is found across most of South Africa, and *C. caffra namaquensis* which has a more limited distribution in northwest South Africa and neighboring Namibia. Our goals are to characterize genetic diversity in South African populations of *C. caffra*, assess genetic discontinuities and connectivity across the landscape, and relate genetic diversity to contemporary and historical environmental features to better understand the processes that underlie patterns of genetic diversity. Although both historical and contemporary landscape structure can have a pronounced influence on population structure, disentangling the effects of historical versus contemporary features on population connectivity, dispersal and migration can be complicated, often requiring a multipronged approach to resolve the temporal effects of a changing landscape (Wang, 2010). Our approach combines predictive species modeling, population genetics, and landscape genetics in combination with two different genetic marker types that have utility at different temporal scales.

We specifically evaluate two primary hypotheses. First, because *C. caffra* is a habitat‐generalist species, we hypothesize that there will be few barriers to dispersal, except for the Nama Karoo aridland biome, from which the species is largely absent (Hockey, Dean, & Ryan, 2005). We hypothesize that the Nama Karoo biome has historically served as a barrier to gene flow between individuals inhabiting the eastern (Grasslands) and western (Fynbos, Succulent Karoo) aridland biomes of South Africa (Figure 1a).

Second, since biomes are not static over time, we hypothesize that demographic and population genetic signatures within *C. caffra* will reflect spatial patterns of dynamic biome change if the historical landscape has been important in structuring genetic diversity. In other words, we expect that stable biome refugia will have high genetic diversity relative to unstable areas and that ephemeral barriers may have left distinct genetic signatures. Based on this framework, we predict that there will be core refugial biome areas for populations inhabiting the Grassland, Succulent Karoo, and Fynbos biomes that will have high genetic diversity. Conversely, in regions where aridland habitat has not been stable, we predict that demographic reconstruction will recover signatures of bottlenecks and expansions that reflect a dynamic historical landscape. We further expect that the signatures of ephemeral barriers will be detectable by using a combination of analytical approaches and markers.

## 2 MATERIALS AND METHODS

### 2.1 Environmental niche models

We used environmental niche models (ENMs) to facilitate the generation of a suite of spatially explicit a priori hypotheses pertinent to the evolutionary history of *C. caffra*. We generated ENMs for the contemporary and recent Pleistocene time periods. Occurrence data were compiled from vouchered museum specimens from our own field research, as well as from records hosted by the Global Biodiversity Information Facility (GBIF; Lane and Edwards, 2007). Models were constructed from 1,100 presence points that spanned nearly the entire distribution of species southern African distribution (Figure S1). Duplicate presence points from the same locality were removed from analyses. Predictive models were built using a modeling approach that performs well with presence‐only data, that is, maximum entropy as implemented in MaxEnt (Phillips & Dudik, 2008). Models were constructed using presence data in conjunction with contemporary climate data from WorldClim (Hijmans, Cameron, Parra, Jones, & Jarvis, 2005) at 30 arc‐second resolution. Pleistocene models were built by projecting the contemporary model onto climates for the last glacial maximum (18–21 kya) and the last interglacial period (120–140 kya) using the general climate models from the DKRZ working group and Otto‐Bliesner, Marshall, Overpeck, Miller, and Hu (2006), respectively. The effect of the regularization constant was evaluated for values ranging from 0.5 to 3, following Warren and Seifert (2011). The response curves were used to assess the fit of the model, and we found smooth response curves with a regularization constant of one, which we used for subsequent models. To avoid overly correlated environmental variables in this study, Pearson's correlation was used to evaluate the relationships among 19 bioclimatic variables (Table S1). Since high correlations were discovered between some variables, the initial set of 19 variables was trimmed to 10 variables (Table S1). Three quarters of the points were used for training, and one quarter of the points were randomly retained to test the model. One hundred replicate bootstrap analyses were generated for each model. The AUC statistic was utilized to assess model performance with scores above 0.90 considered to be excellent (Swets, 1988). Jackknife analyses were used to evaluate the variables with the greatest influence on the contemporary distribution. These models assume that the niche has remained relatively constant and can therefore be projected to differing time periods.

We next built ENMs for each biome and hindcast them as above, except we used the full suite of 19 bioclimatic variables. We used the modern distribution of biomes as input to our models (Mucina & Rutherford, 2005). We identified regions of habitat stability by classifying presence or absence of habitat types in each gridcell for each time period and then used the classified biome layers to partition the landscape into stable (habitat refugia) and unstable (nonrefugial) areas to capture the spatial extent of habitat refugia (e.g., Carnaval et al., 2009; Graham et al., 2006).

### 2.2 Molecular methods

We extracted total genomic DNA using the DNeasy Kit (Qiagen). We genotyped 14 highly polymorphic microsatellite loci for each individual using primers and protocols from Wogan, Feldheim, Voelker, and Bowie (2015). Alleles were assessed using GeneMapper v. 4 (ABI). We tested for linkage disequilibrium and HWE among the microsatellite loci using the log‐likelihood statistic in Genepop v. 4.2 (Rousset, 2008). We PCR‐amplified the entire ND2 mitochondrial gene and used ExoSAP‐IT (Thermo Fisher) to clean the reactions. We then used BigDye chemistry (Thermo Fisher) to cycle sequence the amplicons, followed by a standard ethanol precipitation to remove excess dye; and the Sanger sequenced each fragment in both directions on an ABI 3730. Sequences were edited in Sequencher v. 4 (GeneCodes) and aligned in MAFFT (Katoh & Kuma, 2002).

### 2.3 Population structure

We assessed population structure using our microsatellite data with Structure v. 2.3.4 (Pritchard, Stephens, & Donnelly, 2000). Final Structure analyses were run under the admixture model, testing *k*‐values from 2 to 10 with 20 replicates at each *k* with a run length of 150,000 steps after a 10,000 step burn‐in. These parameters were selected after initial tests of chain convergence; further details are provided in the supplementary documents. To visualize the results, we created a genetic surface from the *q*‐values using IDW interpolation in ArcGIS (ESRI, Redlands) with a one km cell size, a search radius of 12, and a power function of 2 (Latch et al., 2014). Using *k*‐density plots with the Hawths Tools ArcGIS package, we created contour lines at *q* > 0.80 (Latch et al., 2014).

Next, we used discriminant analysis of principal components (DAPCs) which relies on sequential *k*‐means clustering, but does not assume Hardy–Weinberg equilibrium, and has been demonstrated to outperform Structure when populations are hierarchically stratified or when they exchange genes following a stepping‐stone pattern (Jombart, Devillard, & Balloux, 2010). We used DAPC as implemented in the adegenet R package (Jombart, Devillard, Dufour, & Pontier, 2008) and initially retained 50 principal components during the data transformation step. We used a‐score optimization to select the optimal number of principal components (Jombart et al., 2010).

We then used analysis of molecular variation (AMOVA) tests to evaluate the partitioning of genetic variation in *C. caffra* with respect to the two population clustering methods. We compared the partitioning of genetic variation for the populations delimited with DAPC and Structure. We used the sum of squared size differences (R_ST_), and each analysis was run with 16,000 permutations to assess statistical significance of the fixation indices in Arlequin 3.5 (Excoffier & Lischer, 2010).

We calculated the number of haplotypes and a median‐joining haplotype network for our mtDNA marker using PopArt (Leigh & Bryant, 2015), and color‐coded each sample by population.

### 2.4 Population genetics, gene flow, and demography

For the microsatellite (msat) data, we assessed genetic diversity and differentiation among unique geographic localities by calculating Nei's Gst (Nei, 1978), G”st (Hedrick, 2005) and Jost's D (Jost, 2008) using the R mmod package (Winter, 2012). We evaluated global expected versus observed heterozygosity and tested for heterozygote deficiency using a simple *t* test using adegenet. We removed individuals whose genomes were admixed between genetic clusters (e.g., those with <.80 assignment probability), and then used Genepop (Rousset, 2008) and hierfstat (Goudet, 2005) to calculate diversity statistics for each population cluster: allelic richness (using rarefication set to the maximum number of samples in the smallest population), the number of private alleles, *Β* (F_ST_), F_IS_, and Fisher's exact test of genic differentiation. We estimated the mean expected and observed heterozygosity with 1,000 jackknife replicates using the R package PopGenKit (Paquette 2013). We tested for heterozygote deficiency using Genepop. Using the mtDNA data, we calculated the number of pairwise differences (π), nucleotide diversity, and θ_S_ in Arlequin (Excoffier & Lischer, 2010).

We utilized the msat dataset to assess gene flow among the populations in a pairwise manner. To take advantage of the fast mutation rate of the msats, we used two frequency‐based methods: the private allele statistic of Barton and Slatkin (1986) implemented in Genepop, and a Bayesian inference MCMC method implemented in BayesAss, which is able to detect recent gene flow (Wilson & Rannala, 2003). We also used a coalescent‐based estimator to estimate historical gene flow with the program Migrate‐n v.3.6 (Beerli, Mashayekhi, Sadeghi, Khodaei, & Shaw 2019). For BayesAss analyses, we used a MCMC of 1*10^7^ with a 1*10^6^ burn‐in and assessed convergence with Tracer v. 1.5 (Rambaut, Drummond, Xie, Baele, & Suchard, 2018). For Migrate‐n analyses, we used the Brownian stepwise microsatellite mutation model with starting values estimated from F_ST_; the MCMC chain was run 10^6^ steps, with the first 10^4^ discarded as burn‐in. To compare estimated levels of historical and contemporary gene flow, we translated the rates by dividing the *M* (*M* = *m*/*μ*) estimated in Migrate‐n by an estimated average microsatellite mutation rate of 5*10^–4^ (Garza & Williamson, 2001). We then compared the Migrate‐n *m* values with the *m* estimated from BayesAss, thereby enabling us to compare recent and historical rates of gene flow (Chiucchi & Gibbs, 2010).

To evaluate population demography, we first looked for evidence of population bottlenecks by calculating both the original and modified G‐W indexes for each microsatellite locus as part of the *M*‐ratio test (Garza & Williamson, 2001). We then calculated the mean G‐W index in Arlequin. We used the heterozygosity‐excess test (Cornuet & Luikart, 1996) as implemented in Bottleneck v. 1.2.02 (Piry, Luikart, & Cornuet, 1999) to determine whether there is evidence of a population reduction. We used the two‐phase model and set the proportion of multistep mutations to 0.20 (following Peery et al., 2012) with 10,000 iterations to evaluate each microsatellite, and assessed significance using Wilcoxon's one‐tailed test.

We calculated Tajima's D and Fu's F using the mtDNA data to look for signatures of population reduction or expansion, and generated demographic and spatial mismatch distributions with 1,000 bootstrap replicates. We calculated Harpending's R statistic and SSD in which nonsignificant results indicate a failure to reject a model of population expansion. We carried out these analyses for each of the population clusters (admixed individuals removed) in Arlequin. We estimated Bayesian skyline plots (BSP) using Beast v. 1.7.5 (Drummond & Rambaut, 2007). We used the HKY + I + G nucleotide substitution model and enforced a strict molecular clock using the widely applied rates of evolution inferred for the ND2 marker in Hawaiian honeycreepers (Lerner, Meyer, James, Hofreiter, & Fleischer, 2011). We ran the MCMC with 3*10^7^ generations and sampled every 3*10^3^ generations, discarded 10% of the runs as burn‐in, and used Tracer to estimate convergence.

### 2.5 Contemporary spatial patterns of genetic diversity, barrier detection, and connectivity

To assess the spatial–ecological context underlying the contemporary patterns of genetic diversity within *C. caffra*, we used a suite of spatially explicit analyses to investigate patterns of genetic diversity and population connectivity across the landscape. We tested for the null model of isolation by distance using the mantel.randtest command in ade4 (Chessel, Dufour, & Thioulouse, 2004). We next applied a spatial principal components analysis (sPCA) using the spca and global.rtest, and local.rtest functions in the R package adegenet (Jombart, 2008). SPCA can detect cryptic spatial patterns that are not associated with high genetic variation (Jombart et al., 2008; Pavlova et al., 2013). Furthermore, it does not require populations to be in Hardy–Weinberg equilibrium, and it reduces the data into a few axes that best explain the data after accounting for spatial autocorrelation and genetic variation. We estimated a Gabriel graph with the jitter function set to 0.01 to accommodate samples collected from the same localities. We used global and local rtests to assess signal for significant autocorrelation with 10,000 permutations, and then interpolated significant structures across the landscape. Statistically significant global structures reflect positive autocorrelation in the dataset that differentiates between two groups, while statistically significant local structures reflect negative autocorrelation in which neighbors have greater than expected genetic differences (François & Waits, 2016).

To identify potential landscape barriers to gene flow and assess their spatial concordance with habitat features, we employed a barrier detection approach. The Geneland R package (Guillot, Mortier, & Estoup, 2005) appears to be an optimal Bayesian clustering method for barrier detection because it is successful in detecting true barriers under most dispersal distance scenarios (Blair et al., 2012; Safner, Miller, McRae, Fortin & Manel, 2011). We followed recommendations from Blair et al. (2012) with a goal of identifying if barriers exist on the landscape while controlling for the potentially confounding effects of isolation by distance on barrier detection. Using a setting of *k* = 2, we assumed no spatial uncertainty in our points and tested both the correlated and uncorrelated allele frequency models. Analyses used 200,000 iterations with thinning set to 100 and a burn‐in of 25% with five independent runs. We then selected the run with the highest posterior probability and used the cluster memberships to determine barrier location.

To assess whether there are any landscape features that are specifically associated with connectivity and gene flow in *C. caffra*, we used a landscape genetic approach to assess the impact of contemporary landscape features on population connectivity. We constructed friction landscapes using ArcMap 9.3 (ESRI) and then evaluated them in relation to contemporary genetic patterns among *C. caffra* populations. We also implemented an estimated effective migration approach to identify corridors and barriers to gene flow (EEMS: Petkova, Novembre, & Stephens, 2016). Further details can be found in the SOM.

### 2.6 Historical spatial patterns of genetic diversity and connectivity

As we are interested in understanding the historical spatial patterns of genetic diversity across the landscape, we used sPCA (above) to evaluate cryptic genetic diversity in the mitochondrial DNA. We then used the residuals from a linear regression of individual pairwise genetic distances against geographic distances to remove the effects of geographic distance on genetic distance (Manni, Guerard, & Heyer, 2004) by calculating the pairwise genetic distances among all individuals using the K80 model in the function dist.dna in the ape package (Paradis, Claude, & Strimmer, 2004), and great circle distances between each individual using the distm function in the R package geosphere (Hijmans et al. 2005). We used inverse distance weighting (IDW) to build a connection network and then interpolated the residuals of genetic distance in order to identify areas with higher and lower levels of genetic divergence. Areas of unusually high or low genetic divergence, respectively, represent historical barriers or corridors across the landscape. We performed these analyses using the single species genetic divergence tool (power = 2, variable search radius with 12 points) in the Genetic Landscapes GIS Toolbox (Vandergast, Perry, Lugo, & Hathaway, 2010).

### 2.7 Partitioning of genetic variation

We implemented additional AMOVA to evaluate the partitioning of genetic variation with respect to different spatial and taxonomic hypothesis: (A) current biome boundaries; (B) biome stability (two groups: those within stable habitat refugia and those not within refugia); (C) species refugia (two groups: those within predicted species refugia and those not); (D) Geneland detected barrier (2 groups on either side of the putative barrier); and (E) subspecies taxonomy (*C. c. caffra* or *C. caffra namaquensis*). Analyses were carried out as detailed above.

## 3 RESULTS

### 3.1 Environmental niche models

Models had AUC scores above 0.9, suggesting that they had high discriminatory power, and bootstrap replicates were highly consistent across runs indicating nonstochastic results. The present‐day ENM predicts high suitability across most of South Africa for *C. caffra.* Some areas along the western coast and in the Nama Karoo and Savanna biomes are not currently climatically suitable (Figure 1c). Hindcast models for the LGM predict high suitability across much of South Africa, with a northward interior shift, a shift away from the eastern and western coasts, and a greater degree of unsuitable climate in the vicinity of the Nama Karoo biome (Figure 1c). The hindcast LIG model indicates a drastic reduction in suitable climate space throughout the region, with low climate suitability across much of the Succulent Karoo, Nama Karoo, and Savanna biomes (Figure 1c).

Large proportions of the Grassland, Fynbos, and Succulent Karoo biomes are predicted to have remained relatively stable through time (Figure 1b). Large regions of the Savanna and Nama Karoo biomes are predicted to have been unstable. Among the remaining South African biomes, unstable areas were found primarily along biome edges. We identified putative species refugia within the Grassland, Fynbos, and Succulent Karoo biomes in the north near the Namibian border, along the southern coast, and along the border with Lesotho (Figure 1b).

### 3.2 Molecular data

Microsatellite loci have a high mutation rate and capture contemporary processes, while mtDNA has a mutation rate that is roughly four orders of magnitude slower than that of microsatellites (Wang, 2010), and thus should be reflective of historical events. For this study, we genotyped a total of 273 individuals of *C. caffra* from 82 unique South African localities for 14 microsatellite loci. All microsatellite loci were highly polymorphic with the number of alleles ranging from 10 to 33. Evidence of linkage disequilibrium was detected among four locus pairs in these data: CACA12‐CACA34, CACA3‐CNA139, CACA12‐CNA162, and CACA55‐CNA162. We removed CACA34, CNA162, and CACA3 from analyses requiring independence among loci. We also sequenced the entire 1,041 bp ND2 gene for 267 individuals (GenBank Accession numbers MT003298–MT003560). There were 68 variable sites and 33 parsimony informative sites across 54 haplotypes.

### 3.3 Population structure

**Figure 2:**
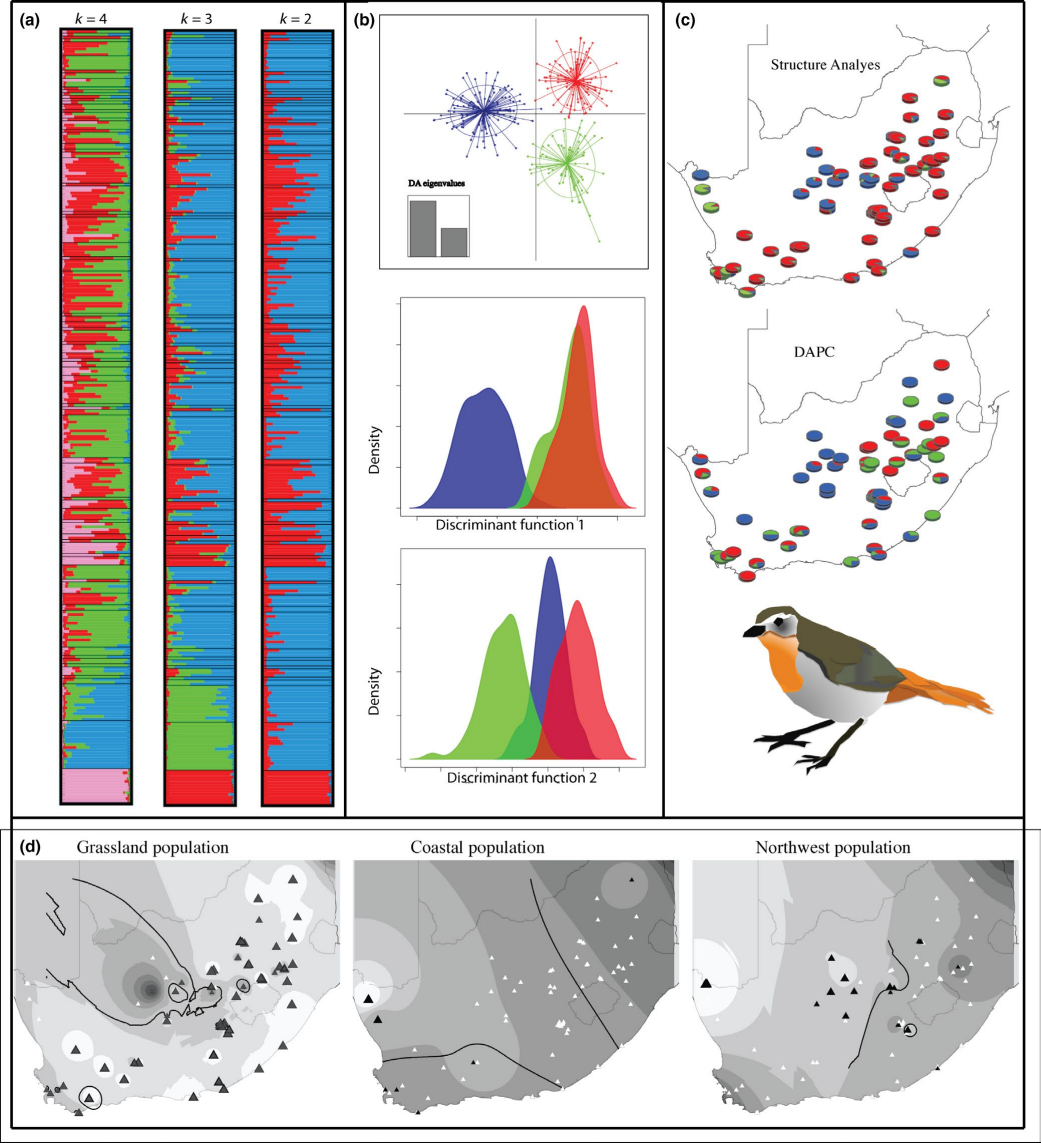
Results from population clustering analyses of *Cossypha caffra* from Structure and DAPC. Both methods delimited three distinct clusters. (a) Structure plots representing *k*‐values from 2 to 4. (b) Results from DAPC analyses. The top panel depicts the clusters delimited by two eigenvalues. The middle and bottom panels show the density distributions for both of the discriminant functions. (c) Maps showing the spatial distribution of clusters and the population composition of each unique locality from Structure analyses (top) and DAPC. (d) Results from Structure analyses depicting the interpolated genetic surfaces (based on *q*‐values) for each of the three populations. The lighter surfaces reflect higher *q*‐values. The black triangles are size‐scaled to reflect the *q*‐value, while white triangles are individuals from the other clusters that did not have mixed ancestry. The dark lines are the 80% contour line for the genetic surface

The number of clusters delineated in Structure analyses recovered a *k*‐value of 3 for uncorrelated models and of 4 for correlated models. Visual inspection of combined‐replicate bar plots indicated that individual assignment probabilities become stable at a *k*‐value of 3 (Figure 2a), and the distribution of q‐matrix values reflects a west‐to‐east cline. Structure identified 110 highly admixed individuals (majority ancestry genotype *q* < 80%). The clusters recovered from Structure analyses consist of one widespread regional cluster spanning 46 unique localities found in the Grassland and Nama Karoo biomes and along the southern coast in the Fynbos biome (“grassland” population hereafter). The other two clusters are comparatively localized with a cluster restricted to two localities along the western coast in the Succulent Karoo biome (“coastal” population), and a cluster found in the northwest near the Namibian border and extending along the Orange River and Vaal River drainages (“northwest” population) (Table 1, Figure 2c,d). DAPC also identified three genetic clusters (Figure 2b). DAPC assigned most individuals with >90% probability to one of the three clusters; only 16 individuals were assigned with <90% certainty; and only nine with <80% certainty. This is likely because DAPC is less sensitive to admixture, since it maximizes the variation present in the dataset and therefore weights the signal of informative ancestry more heavily. The DAPC‐delimited clusters are all widespread and have little geographic structure (Table S2, Figure 2c). At many of the sampling sites, both methods recovered individuals belonging to multiple genetic clusters, indicating that regional populations are not currently spatially segregated (Figure 2c).

**Table 1:** Population metrics for the genetic clusters inferred by Structure analyses

	*N*	# localities	A_R_	A_P_	*Β* (F_ST)_	FIS (id)	FIS (size)	HWE	Mean H_E_ | H_O_
Grassland population	124	46	7.435	111	0.0089	0.1949	0.1704	0.00	0.843 | 0.68
Coastal population	21	2	5.635	8	0.0878	0.1561	0.0757	0.00	0.759 | 0.657
Northwest population	17	3	5.003	6	0.1732	0.1166	0.0977	0.02	0.682 | 0.626

Note

A_R_ is the average allelic richness over all 14 loci for each population. A_P_ is the number of private alleles identified in each cluster. FIS (id) and FIS (size) are estimates of homozygosity or identity. The *p*‐values from the HWE (*U*) test for heterozygote deficiency for each population across all loci. Mean expected and observed heterozygosity (H_E_ | H_O_).

### 3.4 Partitioning of genetic variation

AMOVA analyses revealed that the three recovered DAPC clusters explained just 0.34% of the variance in the dataset, whereas the genetic clusters recovered from Structure analyses explained almost 10% of the variance (Table 2). Since the clusters defined by Structure better maximize between‐group variance relative to within‐group variance, this suggests that they are better able to capture the underlying genetic patterns in our dataset. Hence, all of the remaining analyses used the Structure clusters as the base units.

**Table 2:** Results from AMOVA analyses based on 14 microsatellite loci using R_ST_

Model	Groupings	Pop	Φ_SC_	Φ_ST_	Φ_CT_	% variance among groups
1	DAPC clusters	L	**0.569**	**12.136**	157.047	0.34
2	Structure clusters	L	**17.976**	**11.557**	**154.178**	9.78
A	Contemporary biomes	S	**10.884**	**9.224**	162.822	5.95
B	Biome refugia	S	**18.042**	**8.579**	164.289	9.45
C	Species refugia	S	**−18.734**	**23.841**	163.334	−11.12
D	Geneland barrier	S	**−25.673**	**43.598**	162.961	−14.19
E	Subspecies	S	**−7.665**	**21.293**	163.589	−4.33

Note

The models reflect hypothesized groupings based on genetic clusters, geographic, and morphological partitions. The first two comparisons are of population clustering methods. The remaining AMOVAs (A‐E) evaluate spatial and morphological partitions. Φ_SC_ is variance between groups, Φ_ST_ is variance between populations in a group, Φ_CT_ is variance within populations. In these analyses, populations (Pop) are defined either by unique localities (L), or in accordance with the genetic clusters recovered from Structure (S) analyses. Bold values indicate statistical significance at *p*‐value < .05.

### 3.5 Population genetics and demography

Global estimates of genetic diversity suggest that there are high levels of diversity within *C. caffra* (Tables S3 and S4). We also found a statistically significant global deficit of heterozygotes suggesting genetic structure across the range (*t* = 4.1121, *df* = 13, *p‐*value = .001225). The three population clusters are highly differentiated (Table S5). Fisher's exact test indicated that all loci, except CNA139, were significantly differentiated among the three genetic clusters (Table 3). Furthermore, these clusters retained high levels of genetic diversity and results from Hardy–Weinberg *U* tests for heterozygote deficiency were statistically significant for all but four loci (Table 3), suggesting that there is also some substructuring within clusters. Using rarefication, we found that clusters have similar levels of allelic richness (Table S3). Overall, there are large numbers of private alleles in the data (Table 1), with the mean frequency of private alleles estimated as 0.0267. F_ST_ values that take differences in sampling into account were dramatically different: The grassland population had very low F_ST_ values and high F_IS_ values among subpopulations relative to the other clusters. In contrast, the northwest population recovered high F_ST_ and low F_IS_ values among subpopulations relative to the other clusters. These results are consistent with the grassland population being more homogenous across its wider range as compared to the localized northwest population. F_ST_ values estimated from mtDNA show the greatest differentiation between the coastal and northwest populations (0.11175; Table S6).

**Table 3:** (A) Recent migration rates (*m*) estimated using BayesAss (Wilson & Rannala, 2003) with microsatellite markers. These estimates reflect migration within the past ~5 generations. Confidence intervals are provided below each estimate. (B) Historical migration rates (*m*) estimated using Migrate‐n (Beerli et. al, 2019) with microsatellites. These estimates are calculated by dividing *M* = *m/u* by a standard microsatellite mutation rate of 0.0005 (Garza & Williamson, 2001). Confidence intervals are provided below each estimate. Migration rates should be read from the row population into the column population

	Grassland	Coastal	Northwest
A. Recent			
Grassland	‐	0.0065 (0.0013–0.0117)	0.0035 (0.0001–0.0069)
Coastal	0.0200 (0.0018–0.0382)	‐	0.0147 (0.0006–0.0288)
Northwest	0.0265 (0.0038–0.0492)	0.172 (0.0008–0.0336)	‐
B. Historic			
Grassland	‐	0.0114 (0.0053–0.0237)	0.0286 (0.0008–0.02567)
Coastal	0.0145 (0.0020–0.0210)	‐	0.0069 (0.0–0.4060)
Northwest	0.0172 (0.0053–0.0267)	0.0016 (0.0–0.7822)	‐

The mitochondrial haplotype network recovered 54 haplotypes in the dataset, with one widespread haplotype found throughout the landscape and across populations (Figure S2). Only five haplotypes were recovered for both the coastal and northwest populations, whereas 33 haplotypes were recovered for the grassland population. All three populations have a combination of shared and unique haplotypes. The star‐like shape of the network suggests population expansion, primarily related to the geographically central grassland population.

The number of migrants estimated using Genepop yielded an estimated Nm of 3.76 after size correction, confirming the existence of gene flow among the three regional populations. Estimates of historical and contemporary gene flow using BayesAss and Migrate‐n were generally similar to largely overlapping confidence intervals (Table 3). Our confidence intervals for historic gene flow between the coastal and northwest populations include zero estimates, suggesting a historical absence of gene flow between these clusters. In contrast, contemporary estimates suggest asymmetric gene flow from the northwest into the coastal population (Table 3). Estimates of migration involving the grassland population suggest ongoing low levels of asymmetric gene flow through time, with migration from the coastal and northwest populations into the grassland population remaining steady, and with lower levels of gene flow from the grassland population into the coastal and northwest populations (Table 3).

The G‐W analyses support the existence of population bottlenecks in all three populations, with mean indices of 0.22514, 0.17801, and 0.20637 for the grassland, coastal, and northwest populations, respectively. The coalescent‐based heterozygosity‐excess‐only test detected evidence for population reduction in the coastal population (Wilcoxon 1‐tailed test *p* = .014771). Because the *M*‐ratio reaches equilibrium much slower than does heterozygosity following a bottleneck, in combination, these statistics have frequently been used to infer the relative timing of bottlenecks (e.g., Spear, Peterson, Matocq, & Storfer, 2006; but see Peery et al., 2012). Based on our findings, we can potentially infer that all three populations underwent historical bottlenecks, and the coastal population either is experiencing an ongoing decline, or has recently undergone another bottleneck event.

**Figure 3:**
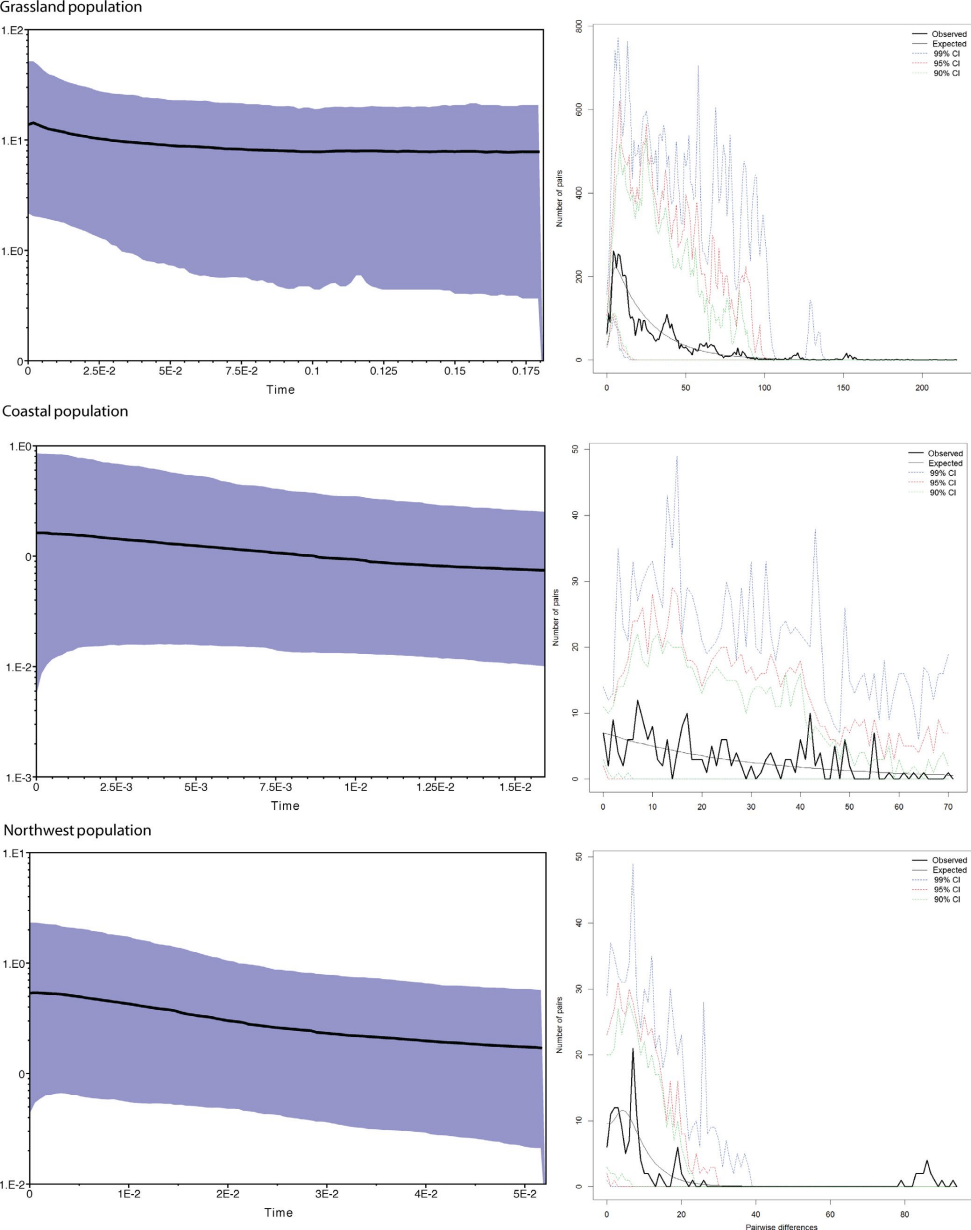
Demographic estimates for each of the three populations. The left panels are Bayesian skyline plots, and the right panels are mismatch distributions. All are estimated from mitochondrial sequence data

Our mtDNA‐based Bayesian skyline plots suggest that the coastal and northwest populations have remained stable or have had a slight population increase over time, while the grassland population has experienced a recent population increase (Figure 3). Tajima's D and Fu's F statistics were negative for all three populations, but only marginally so for the coastal and northwest populations (Table S7). Both statistics provide strong support for a population expansion in the grassland population (Table S7). Mismatch distributions support a population expansion for the grassland population (Figure 3), while both the coastal and northwest were multimodal, which suggests either population substructure or population bottlenecks. SSD and Harpending's R statistics failed to reject demographic or spatial population expansion for any of the populations (Table S7).

### 3.6 Contemporary spatial patterns of genetic diversity and connectivity

**Figure 4:**
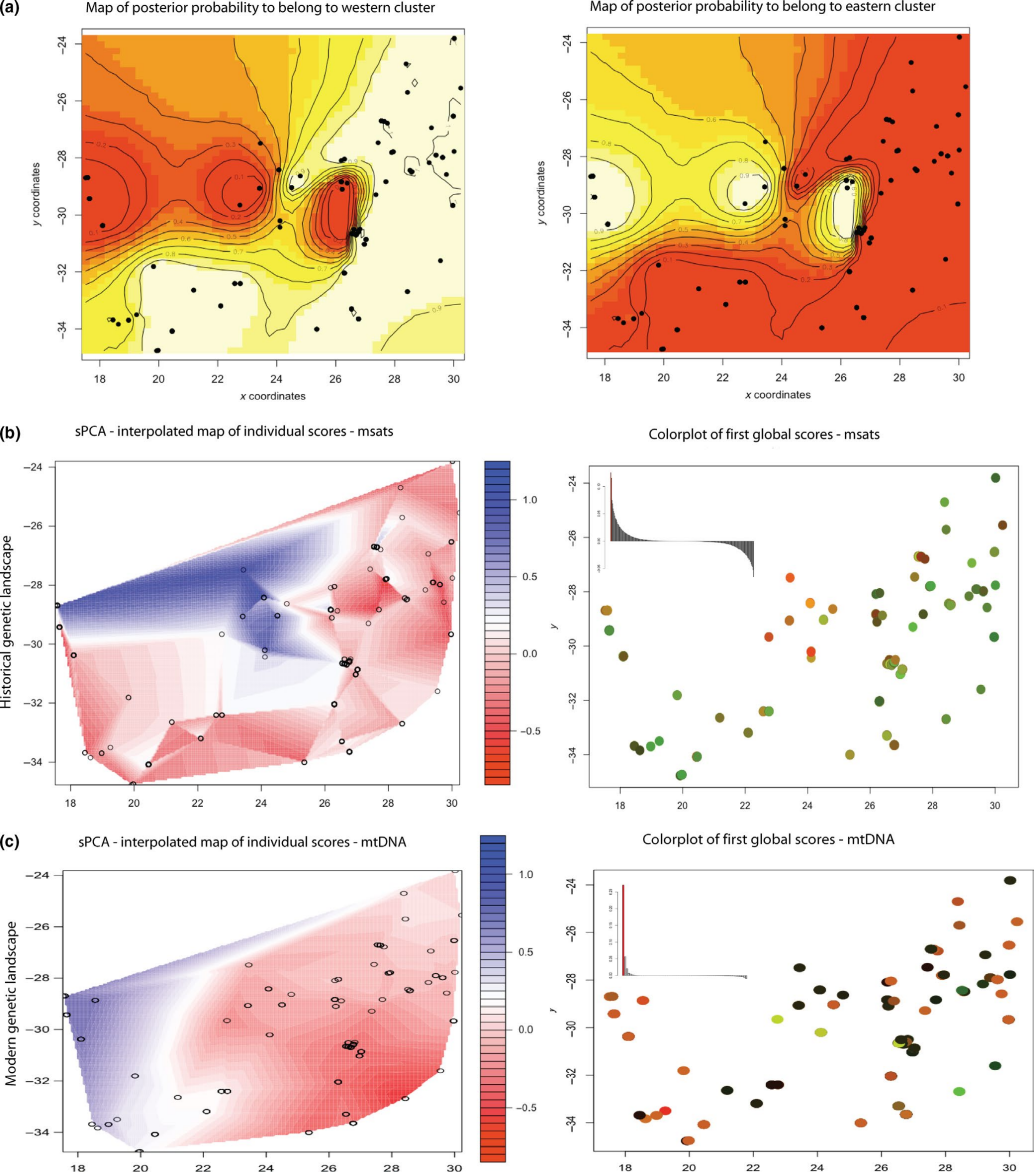
Results from barrier analyses and spatial PCAs of microsatellites and mtDNA. (a) Panels depict the posterior probability of belonging to a western (left) or eastern (right) cluster with a barrier falling within the Nama Karoo biome. (b) The results from sPCAs of microsatellites. The left depicts the interpolated landscape for PC1, while the right provides both a plot of the major global and local structures detected in the data (inset), and a color plot of samples in PC space. (c) The results from sPCAs of mitochondrial DNA. The left depicts the interpolated landscape for PC1, while the right provides both a plot of the major global and local structures detected in the data (inset), and a color plot of samples in PC space

SPCA of the microsatellite data revealed two significant positive eigenvalues (global structure) and no significant negative eigenvalues (local structure) (Figure 4b). PC1 identifies genetic variation corresponding to a northwest‐to‐southeast axis, with the highest levels of genetic differentiation occur in the northern Cape region (Figure 4b), whereas PC2 picks up spatial genetic structure from the northeast coast into the interior (not shown).

Barrier analyses identified a barrier separating the northwestern *C. caffra* populations from the remaining populations (Figure 4a). The barrier is spatially concordant with the Nama Karoo aridland biome, and the results from these analyses are concordant with the structure detected from PC1 of the msat‐based sPCA (Figure 4b). Neither our landscape genetics nor EEMS analyses revealed any contemporary landscape features, barriers, or corridors associated with *C. caffra* gene flow across southern Africa (Table S8, Figure S3).

### 3.7 Historical spatial patterns of genetic diversity and connectivity

**Figure 5:**
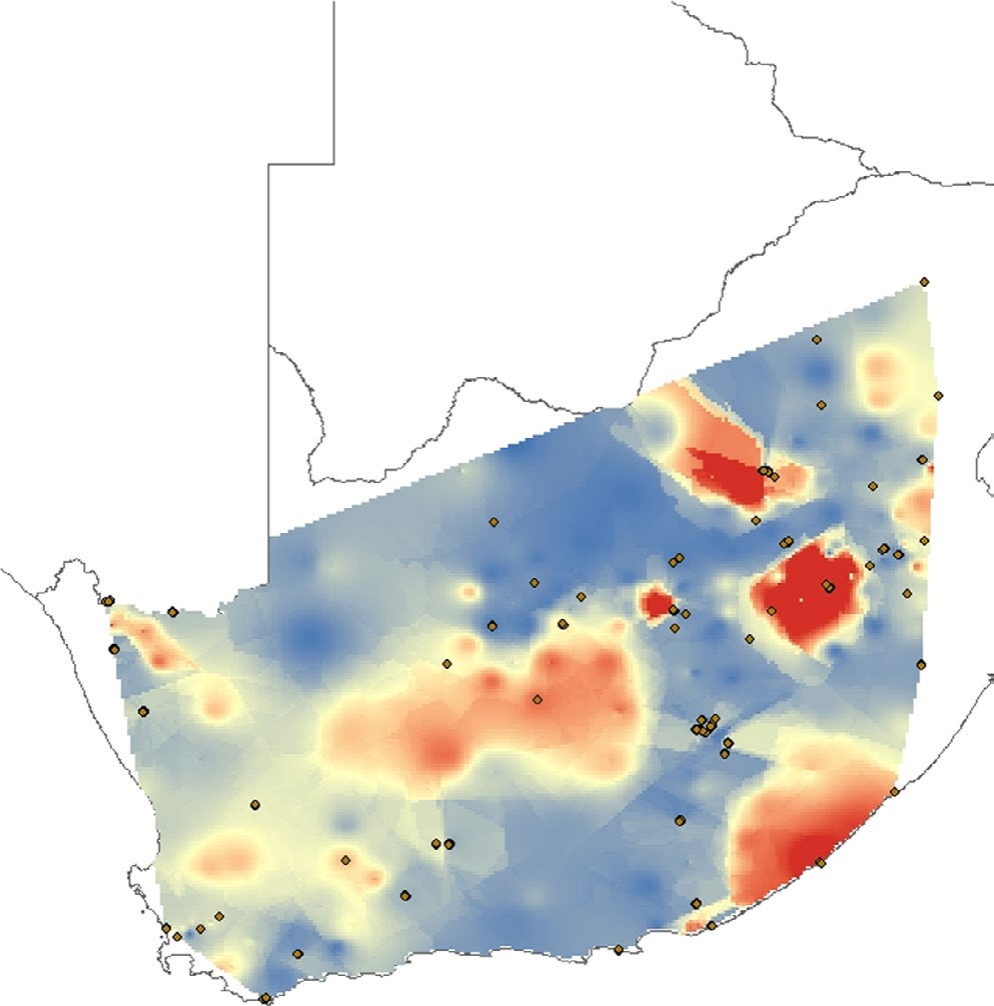
Spatial interpolation of historical residual analyses using mtDNA. Blue areas represent potential corridors where genetic divergence is lower than expected, while red areas represent areas where genetic divergence is higher than expected, and are potential barriers

SPCA of the mtDNA data revealed a single significant positive eigenvalue (global structure) and no significant negative eigenvalues (local structure) (Figure 4c). The sPCA recovered a strong pattern of east‐to‐west differentiation (Figure 4c). Landscape analyses of mtDNA genetic divergence across the subcontinent revealed that the region encompassing the Nama Karoo has historically served as a barrier, with high levels of mtDNA genetic divergence in this region (Figure 5). We also uncovered unusually high levels of observed genetic divergence within the Grassland biome, along the southwestern coast of South Africa, and within the vicinity of Lesotho (Figure 5). These areas fall between regions predicted to have served as species refugia (Figure 1b) within the grassland habitat refuge (Figure 1b).

Of the spatial and taxonomic partitions we implemented in AMOVA, biome refugia explained the greatest amount of variance and maximized the variance among groups relative to variance among populations within groups (Table 2B). Samples from localities in stable habitat refugia versus those in unstable habitats were maximally differentiated. Contemporary biome structure also explained some of the variance in genetic partitioning (Table 2; A). The negative values observed for some of the partitioning strategies (Table 2; E, F, G) reflect a lack of genetic structure with regard to those partitions, including those that should delineate the two Southern African subspecies.

## 4 DISCUSSION

The Cape Robin‐chat is a highly mobile widespread species that inhabits a number of natural and modified habitat types, and is considered a habitat generalist. In South Africa, our data support the presence of three distinct genetic clusters, in contrast to the two named subspecies delineated from morphological data. We find evidence for a widespread group with an extensive range across the Grassland biome, and for two more localized groups: one found primarily in the dry northwest, and a second distributed along the western coastal regions in the Fynbos and Succulent Karoo biomes. The distribution of genetic diversity within *C. caffra* corresponds to boundaries between historically stable aridland biome refugia and areas where habitats have been unstable. Some ecotones between biomes are marked by changes in elevation, for example, the uplifted escarpment separating much the Succulent and Nama Karoo, and very arid regions such as the Knersvlatke, and such physical barriers reinforce biome boundaries. These analyses support a central role for biome history in structuring genetic diversity in *C. caffra.*

The population structure recovered for *C. caffra* is similar to that recovered for other species in South Africa. For example, two major genetic clusters of Smith's Red Rock Rabbit (*Pronolagus rupestris*) exist: one in the northwest and the other in the southeast (Matthee & Robinson, 1996). These clusters are geographically concordant with the northwestern and grassland populations of *C. caffra*. Similarly, genetic structure in the Rock Agama (*Agama atra*) suggests a northwest group that is distinct from a widespread group distributed throughout much of South Africa (Matthee & Fleming, 2002). In both of these examples, Plio‐Pleistocene climate fluctuations and the related formation of habitat refugia are postulated to have driven divergence (Tolley et al., 2014). Likewise, our ENMs recovered potential biome refugia for the Fynbos, Succulent Karoo, and Grassland biomes, and species refugia within these regions, suggesting the existence of persistent centers of habitat and climate suitability for populations of *C. caffra* during the Pleistocene.

The northwest population and the coastal population are separated by only 78 kilometers, but we did not find any identifiable admixed individuals, between the northwest and coastal clusters. The lack of admixed individuals between these two spatially proximate genetic clusters speaks to the potential for some interesting processes at play in this region. At the broad scale of this study, our landscape genetic analyses suggest that contemporary environmental factors are not affecting patterns of gene flow among *C. caffra* populations; however, it is possible that at a localized scale these factors do play a role. The lack of admixed individuals between these two clusters suggests that there may be assortative mating or other intrinsic or extrinsic barriers to gene flow. The isolation of populations in separate refugia may have been of sufficient duration to allow reproductive isolating mechanisms to evolve. Alternatively, in the vicinity of the break between these populations are two potential geographic barriers: the Orange River, which is a barrier for some terrestrial vertebrates (Matthee & Fleming, 2002), but is an unlikely barrier for birds, and the Knersvlatke, which is an expansive rocky flat land that formed during the Miocene when the great western escarpment uplifted (Moon & Dardis, 1988), and which has been subject to Pleistocene marine incursions (Hendey, 1970). The Knersvlatke may be a barrier between the northwest and coastal populations of *C. caffra*, most likely due to the lack of vegetation. Along with *C. caffra*, the Knersvlatke has been found to be a barrier for several reptile and mammal taxa (Matthee & Fleming, 2002; Matthee & Robinson, 1996; Portik et al., 2011; Smit, Robinson, & Vuuren, 2007). Assessing the genetic structure and timing of divergence among the many species that have diverged in the vicinity of the Knersvlatke may provide insight into its role in driving divergence and structuring diversity in western South Africa.

Our findings are consistent with a scenario by which isolated Pleistocene biome refugia have allowed persistence of populations in the central Grassland, in the Fynbos on the southern coast, and in the Succulent Karoo in the northwest. Separating these refugia is the Nama Karoo biome, which has served as a barrier to gene flow for *C. caffra.* The Nama Karoo barrier could have arisen because of habitat instability or habitat unsuitability, or a combination of the two. Indeed, environmental niche models for the Nama Karoo suggest that this biome has not been stable over time (this study, Tolley et al., 2014), and paleoecological evidence provides further support for a highly dynamic landscape (Meadows & Watkeys, 1999). As might be expected, the dynamic nature of the Nama Karoo is reflected in the signatures of demographic expansion and contraction recovered for populations inhabiting this biome (e.g., Barlow et al., 2013). Related is the possibility that the Pleistocene instability of the Nama Karoo biome prevented *C. caffra* population persistence and local adaptation to the arid desert environment such that both instability and unsuitability contribute to the impermeable nature of the Nama Karoo barrier. Among endemic Nama Karoo birds, climate rather than vegetation is the key environmental feature determining bird assemblage patterns (Githaiga‐Mwicigi, Fairbanks, & Midgley, 2002), which suggests that climate‐based barriers may underlie the patterns uncovered in *C. caffra*. Explicit tests of niche divergence or conservatism from *C. caffra* populations from the refugial grassland, coastal, and northwest populations could provide further insight into the basis of the Nama Karoo barrier (e.g., Warren, Glor, & Turelli, 2008).

*Cossypha caffra* populations in the northern part of the Nama Karoo are primarily comprised of individuals from the grassland and northwest populations, whereas those in the southern part of the Nama Karoo are comprised of individuals belonging to the grassland and coastal populations (Figure 2). Furthermore, localities composed of individuals from different genetic clusters exist within the Nama Karoo biome, and suggest that individuals are capable of dispersing across this former barrier; thus, the Nama Karoo has become permeable for *C. caffra*. This has facilitated gene flow. Structure analyses identified numerous admixed individuals (majority ancestry genotype *q* < 0.80), and these admixed individuals are not confined to the contact zones, but instead are scattered among many South African populations. Of the 82 localities we sampled, there were only 14 that did not contain admixed individuals, and these were primarily confined to the grassland population within the Grassland biome, and the northwest. localities at the Nama Karoo and Grassland biome boundary have highly mixed composition and admixed individuals. We find a similar situation where the coastal Fynbos and the Grassland biome meet in the Cape region of South Africa (Figure 2).

Our Migrate‐n results suggest extensive contemporary dispersal and gene flow across the Nama Karoo, and at least some of this dispersal is almost certainly facilitated by the development of human‐modified landscape over the past 200 years (e.g., homesteads; (Hoffman, Cousins, Meyer, Petersen, & Hendricks, 1999; Meadows & Watkeys, 1999)). A second potential conduit creating permeability across the Nama Karoo is the large Orange River and Vaal River drainages. It has been previously postulated that *C. caffra* found in the northwest along the Orange River represent relic populations that are restricted to riverine vegetation (Vernon, 1999). Our findings suggest that the Orange and Vaal rivers may indeed serve as corridors across the arid Karoo connecting the northwest population with the grassland population. Our results are generally consistent with this hypothesis, although we did not recover evidence of historical gene flow. We may simply lack sufficient resolution to detect low levels of historical gene flow. Genome‐wide markers may be needed to better resolve the extent of gene flow along these river drainages.

Our findings of highly admixed individuals and high levels of contemporary gene flow suggest that the population differentiation we identified is disappearing as *C. caffra* populations merge. Although likely quite common, “despeciation,” “ephemeral speciation,” or “speciation in reverse” has only been studied in detail in a handful of species (Block, Goodman, Hackett, Bates, & Raherilalao, 2015; Garrick et al., 2014; Grant & Grant, 2006; Kearns et al., 2018; Ruskey & Taylor, 2016; Seehausen, 2006; Taylor et al., 2006). While many of these examples may have been further along on the speciation continuum (De Queiroz, 2007) than *C. caffra*, most are likewise considered to be “evolutionarily young.” In some of these instances, the loss of distinctive morphotypes provided the first indication that species were merging (Grant & Grant, 2006; Taylor et al., 2006). Quantification of morphological features, particularly plumage traits using museum samples collected over the past century, may similarly reveal changes in *C. caffra* morphology over time.

Voelker et al. (2010) found that Pliocene forest dynamics have had substantial effects on speciation in African forest robins, including members of the genus *Cossypha.* Here, we have shown that aridland refugia have played an important role in structuring within‐species genetic diversity in the South African Cape Robin‐chat (*C. caffra*). This finding supports spatial analyses suggesting that aridland biome refugia are important in structuring avian diversity in South Africa, and this is the first study to confirm this using genetic approaches.

## CONFLICT OF INTEREST

The authors declare no competing financial interests in relation to this work.

## AUTHOR CONTRIBUTIONS

GOUW designed the study, collected the data, analyzed the data, and wrote and edited the manuscript. GV designed the study, collected the samples, and edited the manuscript. GO collected the samples and edited the manuscript. RCKB designed the study, collected the samples, and wrote and edited the manuscript.

## Supporting information

Supplementary Material

Appendix S1

## Data Availability

Sequence data have been deposited under GenBank Accession Numbers (MT003298‐MT003560). The microsatellite genotypes are deposited as supplemental data to this study (Appendix S1).
