# Supplementary material for: Biome stability predicts population structure of a southern African aridland bird species
Source: Ecol Evol. 2020 Mar 27;10(9):4066–81. doi: 10.1002/ece3.6175 (PMC7244808; doi:10.1002/ece3.6175)
Supplement: Supplementary file 1 — Supplementary Material [file ECE3-10-4066-s001.pdf]

SOM: Wogan et al. *Cossypha caffra*

Detailed Methods & Results

Landscape Genetics Analyses: Since the species distribution models were built using climate data and reflect suitable climate space for *C. caffra*, we used the inverse of the suitability score generated from the contemporary species distribution model as a resistance layer (Richards *et al*. 2007). South Africa has a strong east-west aridity gradient, and in the more arid parts of the range*, C. caffra* populations are often found along waterways, so we evaluated two environmental variables relating to water; aridity and hydrology. For aridity we calculated an aridity index (Q) following Guillaumet *et al*. (2008), and then reclassified this from 0-2 to create a cost matrix. For hydrology we created a cost matrix based on waterways, with permanent waterways assigned a lower friction score than intermittent waterways. Finally, to explicitly evaluate the role of biomes in structuring genetic diversity in *C. caffra*, we used the modern distribution of biomes (Mucina and Rutherford 2005) and coded each biome facilitating or impeding gene flow; e.g. biomes with open habitats supporting tree and shrubs facilitate connectivety, while both open habitats lacking tree and shrub cover (e.g. the Nama Karoo) and closed canopy habitats (e.g. forest) impede connectivity. For each cost matrix we conducted a Mantel and partial Mantel test (controlling for geographic distance) to relate least cost path distances to linearized Nei's Gst between sampling sites. Since there are potential problems that arise when using Mantel and partial Mantel tests (Guiilot & Rousset 2013), we also used multiple matrix regressions with randomization (MMRR) (Wang 2013) which iteratively measures the relationship between genetic distance and one matrix while holding the remaining matrices constant and assess significance through

random permutations to generate a null distribution. Analyses were performed for the complete microsatellite dataset (e.g. all individuals regardless of genetic cluster).

Structure analyses: We assessed population structure using our microsatellite data with Structure v.2.3.4 (Pritchard *et al*. 2000). Structure analyses were run under the admixture model, testing k-values from 2 to 10 with 20 replicates at each k with a run length of 150,000 steps after a 10,000 step burn-in; further details are provided in the supplementary documents. We ran replicate analyses using the independent and correlated allele frequencies options to assess if accounting for shared ancestry among populations improved inference (e.g., Falush *et al*. 2003; Rosenberg *et al*. 2005). To estimate k we employed the Evanno deltaK method (Evanno *et al*. 2005) implemented in StructureHarvester (Earl and vonHoldt 2012), this k-value was used as kmax. We then followed these analyses with CLUMPP (Jakobsson and Rosenberg 2007) and DISTRUCT (Rosenberg 2004) to combine replicate runs and visualize assignment probabilities for all values of k less than or equal to kmax. We ran multiple independent replicates under the same conditions and assessed k for each to ensure that our estimates of k were robust.

EEMS Analyses: These analyses are based on a stepping-stone model, and use circuit theory to integrate across all possible migration rates between any two individuals, and generates an expected genetic dissimilary, which can then be interpreted as an estimated effective migration surface on the landscape (Petkova *et al*. 2016). We tested a number of grid sizes (10, 50, 100 demes) to ensure that our results are independent of grid size, and

obtained similar inferred EEMS, thus we present the one based on a grid size of 50. All analyses were carried out with 2 x10^6 MCMC iterations, with 1 x10^6 step burn-in, and with 9999 thinning iterations. We then constructed plots using the rEEMSplots package in R (Petkova *et al*. 2016).

We found that the EEMS analyses were consistent with an overall pattern of isolation by distance. Least cost path analysis using G_{ST} and Mantel tests recovered a statistically significant association between geographic distance and genetic distance for the complete dataset. None of the remaining matrices we tested had a statistically significant correlation with genetic structure (Table S8). After controlling for geographic distance, we did not find evidence for any significant associations between the variables we tested and genetic distance among either the complete dataset or the genetic clusters identified from population structure analyses. Multi-matrix regression did not recover any significant correlations between genetic data and geographic distance or contemporary environmental features. (Table S8).

References

Earl DA, vonHoldt BM (2012). Structure Harvester: a website and program for visualizing Structure output and implementing the Evanno method. *Conservation Genetics Resources* **4:** 359-361.

Evanno G, Regnaut S, Goudet J (2005). Detecting the number of clusters of individuals using the software Structure: a simulation study. *Molecular Ecology* **14:** 2611-2620.

Falush D, Stephens M, Pritchard J (2003). Inference of population structure using multilocus genotype data: Linked loci and correlated allele frequencies. *Genetics* **164:** 1567-1587.

Jakobsson M, Rosenberg NA (2007). CLUMPP: a clustering matching and permutation program for dealing with label switching and multimodality in analysis of population structure. *Bioinformatics* **23**(14)**:** 1801-1806.

Mucina E, Rutherford MC (2005). The vegetation of South Africa, Lesotho and Swaziland. SANBI, Pretoria.

Petkova D, Novembre J, Stephens M (2016). Visualizing spatial population structure with estimated effective migration surfaces. *Nature Genetics* **48**(1)**:** 94-100.

Pritchard J, Stephens M, Donnelly P (2000). Inference of population structure using multilocus genotype data. *Genetics* **155:** 945-959.

Rosenberg NA (2004). Distruct: a program for the graphical display of population structure. *Molecular Ecology Resources* **4**(1)**:** 137-138.

Rosenberg NA, Mahajan S, Ramachandran S, Zhao C, Pritchard J, Feldman MW (2005). Clines, clsuters, and the effect of study design on the inference of human population structure. *PLoS Genetics* **1**(6)**:** e70.

Figure S1. Map depicting the distribution of *Cossypha caffra* across southern Africa. Blue dots present populations sampled for genetic analyses. Purple dots represent 1100 presence points used in the species distribution models.

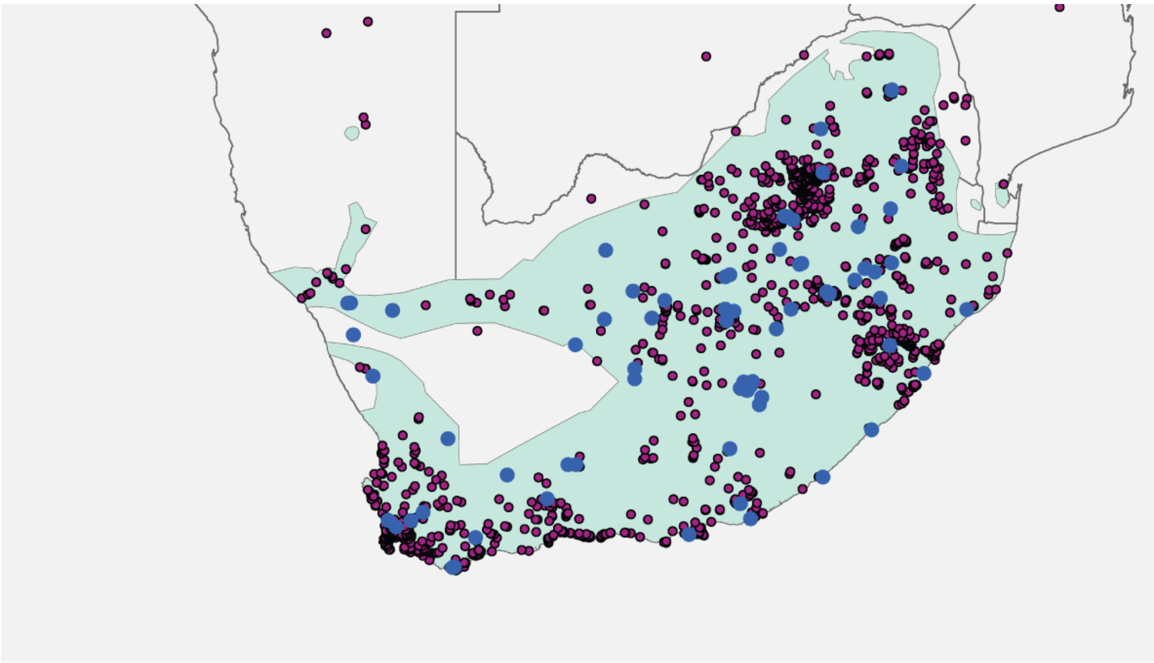

Figure S2. Haplotype network of ND2 mtDNA color coded by population.

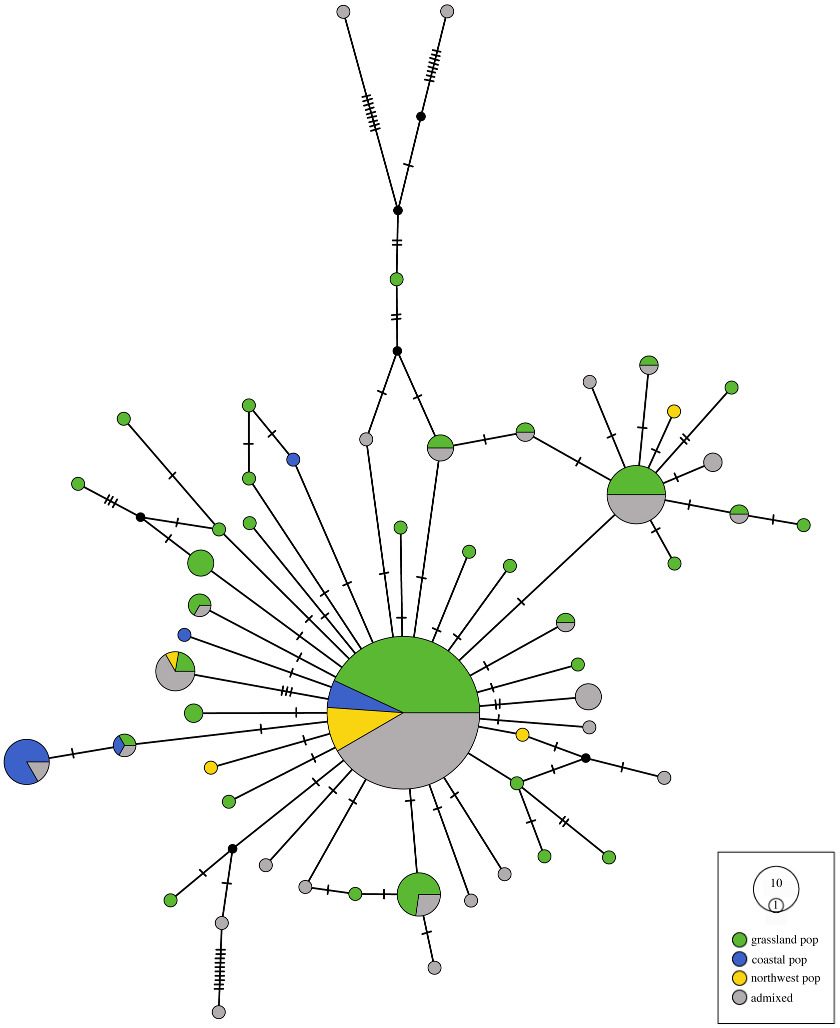

Figure S3. Results from EEMS analyses

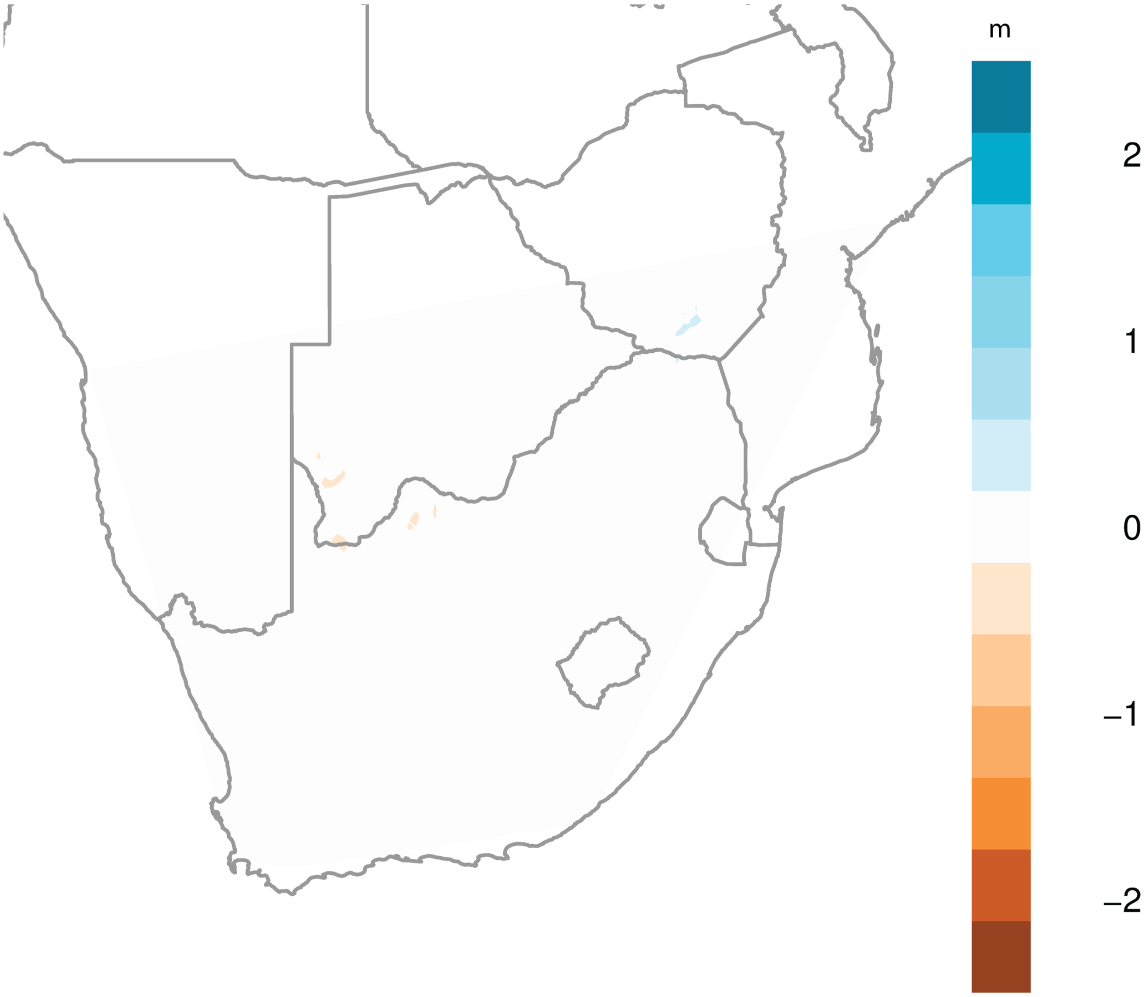

Table S1. The 19 Bioclim variables used in this study (obtained from WorldClim at 30 arc second resolution). Variables used for final analyses for species environmental niche models are indicated in bold.

**Bioclim 1**	Annual mean temperature
Bioclim 2	Mean diurnal range
Bioclim 3	Isothermality
**Bioclim 4**	Temperature seasonality
Bioclim 5	Max temperature of warmest month
Bioclim 6	Min temperature of coldest month
Bioclim 7	Temperature annual range
**Bioclim 8**	Mean temperature of wettest quarter
**Bioclim 9**	Mean temperature of driest quarter
**Bioclim 10**	Mean temperature of warmest quarter
Bioclim 11	Mean temperature of coldest quarter
**Bioclim 12**	Annual precipitation
Bioclim 13	Precipitation of wettest month
Bioclim 14	Precipitation of driest month
**Bioclim 15**	Precipitation seasonality
Bioclim 16	Precipitation of wettest quarter
**Bioclim 17**	Precipitation of driest quarter
**Bioclim 18**	Precipitation of warmest quarter
**Bioclim 19**	Precipitation of coldest quarter

Table S2. Population metrics for the genetic clusters inferred by DAPC. A_R is the average allelic richness over all 14 loci for each population. A_P is the number of private alleles identified in each cluster. FIS (id) and FIS (size) are estimates of homozygosity or identity. The p-values from the HWE (U) test for heterozygote deficiency for each population across all loci. Mean expected and observed heterozygosity (HE | HO).

DAPC (k=3)	n	# localities	A_R	A_P	*B* (F_{ST})	FIS (id)	FIS (size)	HWE	Mean H_E | H_O
Population 1	109	47	13.912	29	0.0482	0.1961	0.1428	0.00	0.817 | 0.656
Population 2	64	42	13.604	13	0.0372	0.1935	0.1035	0.00	0.824 | 0.666
Population 3	91	40	13.936	21	0.0586	0.1973	0.2584	0.00	0.807 | 0.648

Table S3. Allelic richness for each allele for each population.

Locus	Structure (k=3)		
	grassland population	coastal population	northwest population
CACA3	5.572372	2.998912	3.727263
CACA12	7.321552	5.762033	5.731225
CACA26	8.897643	6.955028	6.320711
CACA27	6.419660	4.380795	3.889423
CACA34	7.109831	7.120889	5.372645
CACA43	7.231372	6.334116	6.541199
CACA55	7.282387	5.549322	6.806452
CACA56	10.836551	8.491743	6.802725
CACA66	6.749343	3.927957	5.114634
CACA78	11.162053	8.383714	6.659871
CNA139	4.636522	3.370335	3.438869
CNA162	9.248333	5.709758	3.638999
CNA233	5.462098	3.840216	4.000000
CNA142	6.160189	6.063262	1.994084

Table S4. Global and population genetic diversity metrics for *Cossypha caffra.*

	Hs	Ht	Nei's Gst	Hedrick's G"st	Jost D_het
Global	0.78871523	0.84628572	0.06802726	0.32576456	0.27592729
grassland pop	0.79249405	0.84544251	0.06262809	0.30809167	0.26083631
coastal pop	0.75814804	0.77298700	0.01919691	0.15575915	0.12271110
northwest pop	0.9726290	0.6919522	-0.4056305	-27.8851442	-15.3818277

Table S5. The total number of alleles per microsatellite locus used in this study and the p-values from Fisher's exact probability test of genic differentiation among the three populations of *Cossypha caffra*, and from the Hardy-Weinberg U test of heterozygote deficiency.

Locus	Total Number Alleles	Fisher's Exact Test (p-value)	Hardy-Weinberg (U test) (p-value)
CACA3	13	**0.0000**	**0.0000**
CACA12	14	**0.0000**	**0.0000**
CACA26	21	**0.0000**	**0.0000**
CACA27	33	**0.0000**	**0.0066**
CACA34	13	**0.0000**	**0.0077**
CACA43	27	**0.0000**	0.0930
CACA55	24	**0.0000**	**0.0000**
CACA56	27	**0.0000**	0.6214
CACA66	17	**0.02059**	**0.0004**
CACA78	31	**0.0000**	0.2213
CNA139	10	0.15398	**0.0000**
CNA162	18	**0.0000**	**0.0000**
CNA233	10	**0.0000**	**0.0000**
CNA142	12	**0.0000**	0.2152

Table S6. F_{ST} based on pairwise differences from mtDNA above the diagonal for *Cossypha caffra* populations. Statistically significant F_{ST}-values are in bold.

	grassland	coastal	northwest
grasslands	0	0.02835	0.03206
coastal	-	0	**0.11715**
northwest	-	+	0

Table S7. Population diversity metrics and demography calculated from mtDNA for each of the populations. Statistically significant values are indicated in bold.

population	n	Pairwise differences (Pi)	Nucleotide Diversity	Theta (S)	Tajima's D	Fu's F	Demographic SSD	Demographic Harpending's R	Spatial SSD	Spatial Harpending's R
grassland	113	29.156	0.028	17.547	**-2.326** (0.000)	**-23.272** (0.002)	0.001 (0.970)	0.001 (1.000)	0.001 (1.000)	0.001 (1.000)
coastal	21	22.781	0.022	2.502	-1.003 (0.187)	**-7.272** (0.007)	0.0100 (0.840)	0.019 (0.490)	0.009 (0.940)	0.0188 (0.510)
northwest	17	16.118	0.015	6.507	**-1.915** (0.014)	**-6.485** (0.007)	0.0189 (0.600)	0.026 (0.690)	0.0187 (0.670)	0.015 (0.720)

Table S8. Results from statistical tests assessing associations between contemporary environmental features and genetic diversity. The left column provides the results from Mantel and partial Mantel tests. The r^2 value followed by its p-value are given for each test. Mantel tests are on the top line of each comparison and the partial Mantel controlling for geographic distance is on the bottom line. The right column provides the regression coefficients and significance obtained from MMRR analyses. Significant values at $p < 0.05$ are in bold.

	Nei's Gst	MMRR
Euclidean Distance	0.1151 I **0.041096**	0.0165 I 0.3599
Contemporary Biomes	-0.07271 I 0.77492<br>-0.07577 I 0.78782	0.0712 I 0.3903
Contemporary Climate	0.09957 I 0.089991<br>-0.01693 I 0.59944	0.0046 I 0.9394
Contemporary Aridity	-0.01321 I 0.56914<br>-0.09324 I 0.85091	-0.0002 I 0.9979
Waterways	0.1083 I 0.067493<br>0.01133 I 0.44006	-0.0225 I 0.6303
